# Mid-term Survivorship and Functional Outcomes of Metaphyseal Sleeves for Addressing Anderson Orthopaedic Research Institute (AORI) Type 2 and 3 Bone Defects in Revision Total Knee Arthroplasty

**DOI:** 10.7759/cureus.69381

**Published:** 2024-09-13

**Authors:** Kushal Hippalgaonkar, Kikkuri Rajeev Reddy, Praharsha Mulpur, Tarun Jayakumar, Swapnil Joshi, A.V. Gurava Reddy

**Affiliations:** 1 Orthopaedics, KIMS-Sunshine Hospitals, Hyderabad, IND

**Keywords:** bone loss, constrained liner, metaphyseal sleeves, revision total knee arthroplasty, zonal fixation

## Abstract

Introduction

Revision Total Knee Arthroplasty (RTKA) is a complex procedure challenged by significant bone loss, necessitating effective restoration techniques. This study investigates the clinical outcomes and complications of metaphyseal sleeves in RTKA with severe metaphyseal bone loss, aiming to evaluate their efficacy over a minimum four-year follow-up.

Methods

This was a retrospective observational study on 29 patients who underwent RTKA with Anderson Orthopaedic Research Institute (AORI) type II or III bone defects using porous coated tibial and/or femoral metaphyseal sleeves from December 2016 and January 2019. Data collection included demographic information, etiology for revision, and functional outcomes assessed by the Knee Society Score (KSS) and Oxford Knee Score (OKS). Statistical analysis and Kaplan-Meier survival analysis were performed.

Results

The cohort comprised patients with a mean age of 62.6 years (SD=7.8), predominantly female (N=21, 71.4%). The primary indication for RTKA was aseptic loosening (N=18, 62.1%) followed by Prosthetic Joint Infection (PJI). Significant improvements were noted in the range of motion. Both KSS (Pre-op:57.97 vs. Post-op:73.59) and OKS (Pre-op:15.86 vs. Post-op:30.66) showed highly significant improvement (p<0.0001). Radiographic assessments indicated stable component position and signs of osseous integration without any osteolysis. No sleeve-related complications were observed. Survival analysis demonstrated a high cumulative survival probability over the study period.

Conclusion

Metaphyseal sleeves offer a viable solution for managing severe bone loss in RTKA, providing stable fixation, restoring joint line kinematics, and facilitating stress distribution to the metaphyseal region. This study corroborates the effectiveness of metaphyseal sleeves in challenging revision scenarios, aligning with previous research.

## Introduction

Revision total knee arthroplasty (RTKA) is increasingly significant in orthopaedic surgical practice, posing a substantial challenge in addressing bone loss. The etiology of RTKA encompasses various factors, including osteolysis, aseptic loosening, chronic periprosthetic joint infection (PJI), component subsidence, and polyethylene insert wear [1]. Effective management of bone loss in RTKA necessitates the restoration of joint line kinematics and the achievement of stable implant fixation [2]. The Anderson Orthopaedic Research Institute (AORI) classification is utilized to categorize bone loss, and there exists a plethora of options for reconstructing bone defects [3]. These include the use of cement, screws with cement, bone grafts, metal augments, and sleeves or cones for metaphyseal defects and fixation [4-8].

Introduced in the late 1970s, metaphyseal sleeves have been increasingly employed in RTKA. These modular cementless metaphyseal fixations have demonstrated encouraging short-term outcomes. Metaphyseal sleeves leverage Wolff's law to induce bone ingrowth toward the sleeve, thereby distributing stress to the metaphysis and provide stable zone 2 fixation. This process enhances the fit at the metaphyseal and diaphyseal levels and mitigates stress risers in the presence of bone. The sleeves have a Morse taper junction that connects them to the tibia or femoral component. These sleeves are coated with titanium beads and act as structural allografts, helping to transfer the load to the metaphyseal region. Successful osseointegration of these sleeves is associated with better long-term implant survival, improved restoration of the joint line, and excellent torsional stability, especially for cases with severe bone loss [9].

For severe bone loss accompanied by instability, the use of constrained prostheses should be contemplated. Revision surgery failures can be attributed to the employment of either excessive or insufficient constraint. Nonetheless, research on metaphyseal sleeves is limited, featuring small patient cohorts and a lack of extensive data on functional outcomes.

This study aimed to assess the clinical and radiological outcomes of metaphyseal sleeves in the management of severe metaphyseal bone loss during RTKA, with a minimum follow-up period of four years.

## Materials and methods

This was a retrospective observational study, sanctioned post-approval from the institutional ethical committee (SIEC/2022/492) along with informed consent from all patients. We aimed to evaluate the outcomes of patients who underwent revision total knee arthroplasty (RTKA) utilizing sleeves, from December 2016 to January 2019, by analyzing our hospital's patient registry. The primary indications for undertaking RTKA encompassed aseptic loosening, instability, and peri-prosthetic joint infection (PJI). Inclusion criteria were patients exhibiting AORI type II or III bone defects. Specifically, for PJI cases, only patients who had undergone a two-stage revision process were considered eligible, while those receiving DAIR (Debridement, Antibiotics with Implant Retention) protocols were excluded from the study. Patients were excluded if they had incomplete pre-operative and post-operative functional scores and clinical data, unknown implant sizes, and incomplete, missing, or inadequate demographic and surgical data.

During RTKA procedures, patients were fitted with press-fit tibial and/or femoral porous-coated metaphyseal sleeves. Data collected included patient demographics including age, gender, weight, height, Body Mass Index (BMI), American Society of Anesthesiologists (ASA) grading, and Charlson Comorbidity Index (CCI). Functional outcomes were measured using the Knee Society Score (KSS) and Oxford Knee Score (OKS) until the final follow-up. The extent of intraoperative bone loss was evaluated using the AORI system, where Type II defects indicated a significant metaphyseal bone loss in femoral/tibial condyles, and Type III defects involved a critical metaphyseal segment loss and collateral ligament detachment, often requiring more constrained revision components.

Surgical technique

Exposure for RTKA was performed via a standard medial parapatellar approach in all cases, with subsequent removal of synovium and scar tissue in the knee joint. In cases with limited exposure or difficulty in everting the patella, a quadriceps snip was performed. In cases of revision for aseptic loosening or instability, primary components were removed with the help of an oscillating saw or osteotomes. In the second-stage procedure for PJI, the antibiotic-laden cement spacer was removed before re-debridement.

All patients received the Sigma TC3 Revision Knee Systems (DePuy Synthes, Warsaw, USA) as part of their revision TKA. The medullary canals of the femur and tibia were located and reamed until adequate diaphyseal fit was achieved. The metaphysis was then prepared for porous coated sleeve by sequentially reaming and broaching to accommodate. The appropriate size of the sleeve was determined based on its fit within the metaphysis, without any toggle or play in any direction, and also based on the restoration of the joint line. Larger sleeves can allow for the distal placement of the femoral component, along with the use of femoral augments, to reduce the extension gap. After trialling, the final prosthesis was assembled and cemented using Palacos R+G antibiotic cement (Heraeus Medical, Hanau, Germany). The smallest metaphyseal sleeve (Size 29) is cemented, whereas the larger sizes are all porous coated, and it is ensured that cement is not applied over the porous surface of the sleeve. The standard revision technique was employed to achieve optimal alignment, restore the joint line, balance the flexion-extension gap, track the patella, and improve the range of motion. The rehabilitation process adhered to the same protocols as those for primary total knee arthroplasty (TKA), ensuring a successful outcome.

**Figure 1:**
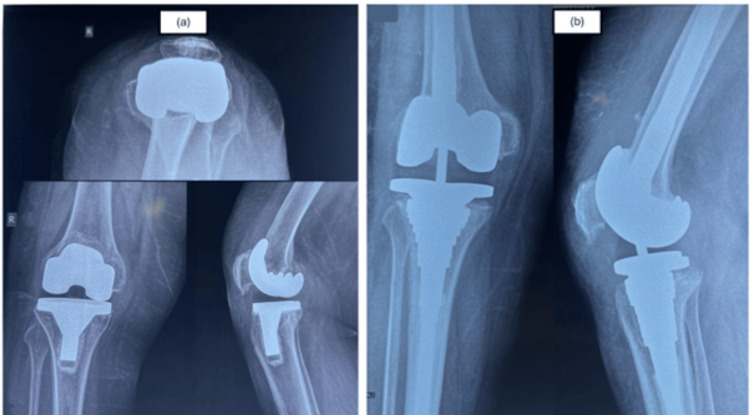
(a) Pre-operative radiographs of Anteroposterior (AP), Lateral, and Skyline view of the right knee showing Type IIB AORI tibial defect along with polyethylene wear. (b) AP and lateral radiographs at final follow-up of 5 years showing well-fixed metaphyseal sleeve and stem construct without any signs of loosening. AORI: Anderson Orthopaedic Research Institute

Follow-up evaluations were conducted at 1 month, 3 months postoperatively and annually thereafter for a minimum 4 years follow-up duration. Knee function was assessed using the KSS [10] and the patient-reported OKS. Serial radiographic assessment was performed at all follow-up visits to monitor for any signs of component failure or bone integration issues (Figure 1).

Statistical analysis

The data were processed using SPSS Version 24 (IBM, Armonk, USA). Continuous variables were reported as means along with their standard deviations and analyzed using student t-test, while categorical variables were depicted through frequencies and percentages and analyzed using chi-square test. Kaplan-Meier survival analysis was employed to assess survival rates.

## Results

Out of the initial pool, 29 patients met the eligibility criteria and were included in the final assessment, having been followed for a minimum of 4 years after undergoing revision total knee arthroplasty using sleeves. Patients had a mean age of 62.6 years (Range: 50-75 years) at the time of surgery with the majority being females (N=21, 72.4%). Patient demographics are summarised in Table 1.

**Table 1:** Demographic characteristics of the patient population ASA- American Society of Anesthesiologists, CCI- Charlson Comorbidity Index

Parameter	Mean	SD
Age	62.62	7.86
BMI	29.98	5.51
	Number	Percentage
Gender		
Female	21	72.4
Male	8	27.6
Side		
Left knee	17	58.6
Right knee	12	41.4
ASA grade		
ASA 1	4	13.8
ASA 2	22	75.9
ASA 3	3	10.3
CCI		
Mild	8	27.59
Moderate	16	55.17
Severe	5	17.24

Etiology for revision

**Figure 2:**
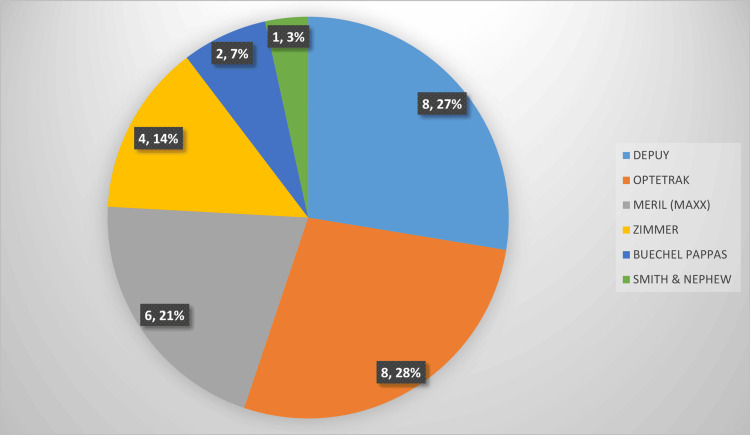
Primary implant manufacturer details DePuy Synthes, Warsaw, USA; Optetrak, Gainesville, United States; Meril (Maxx), Vapi, India; Zimmer Biomet, Warsaw, United States; Buechel Pappas, TTK Healthcare, Chennai, India; Smith & Nephew, London, United Kingdom

The underlying cause for the primary total knee arthroplasty (TKA) was mainly primary osteoarthritis, accounting for 26 cases (89.7%). Primary TKA implant manufacturer details are summarized in Figure 2.

The predominant reason for the revision was aseptic loosening, identified in 18 cases (62.1%), making it the most frequent cause. This was followed by stage-2 revision for PJI in seven cases (24.1%) and instability in three cases (0.3%). The average duration from the index surgery to the point of revision was 81.38 months (SD=31.9) (Table 2).

**Table 2:** Primary etiology and prior surgical details

	Mean	SD
Time from index surgery (months)	81.38	31.899
	Number	Percentage
Primary Etiology		
Osteoarthritis	26	89.7
Rheumatoid arthritis	3	10.3
Cause of Revision		
Aseptic loosening	18	62.1
Infection	7	24.1
Instability	3	10.3
Periprosthetic fracture	1	3.4
Previous revision surgeries		
No	17	58.6
Yes	12	41.4

The DePuy Sigma TC3 Revision Knee system was utilized in all patients. Tibial metaphyseal sleeves were predominantly employed, with the 29 mm sleeve being the most frequent choice, used in 48.3% (N=14) of the cases, followed by the 37 mm sleeve in 41.4% (N=12) of the cases. Femoral sleeves were implemented in only three patients (10.3%), with the 31 mm size used in all of them. Additional implant details are provided in Table 3.

**Table 3:** Revision implant details - Sigma TC3 Revision Knee Systems (DePuy, Warsaw, USA)

Implant Details	Number	Percentage
Femur sleeve		
31 mm	3	10.3
Tibia sleeve		
29 mm	14	48.3
37 mm	12	41.4
45 mm	3	10.3
Insert sizes		
10 mm	7	24.1
12.5 mm	7	24.1
15 mm	2	6.9
17.5 mm	5	17.2
20 mm	4	13.8
22.5 mm	4	13.8

Clinical and radiological outcomes

**Figure 3:**
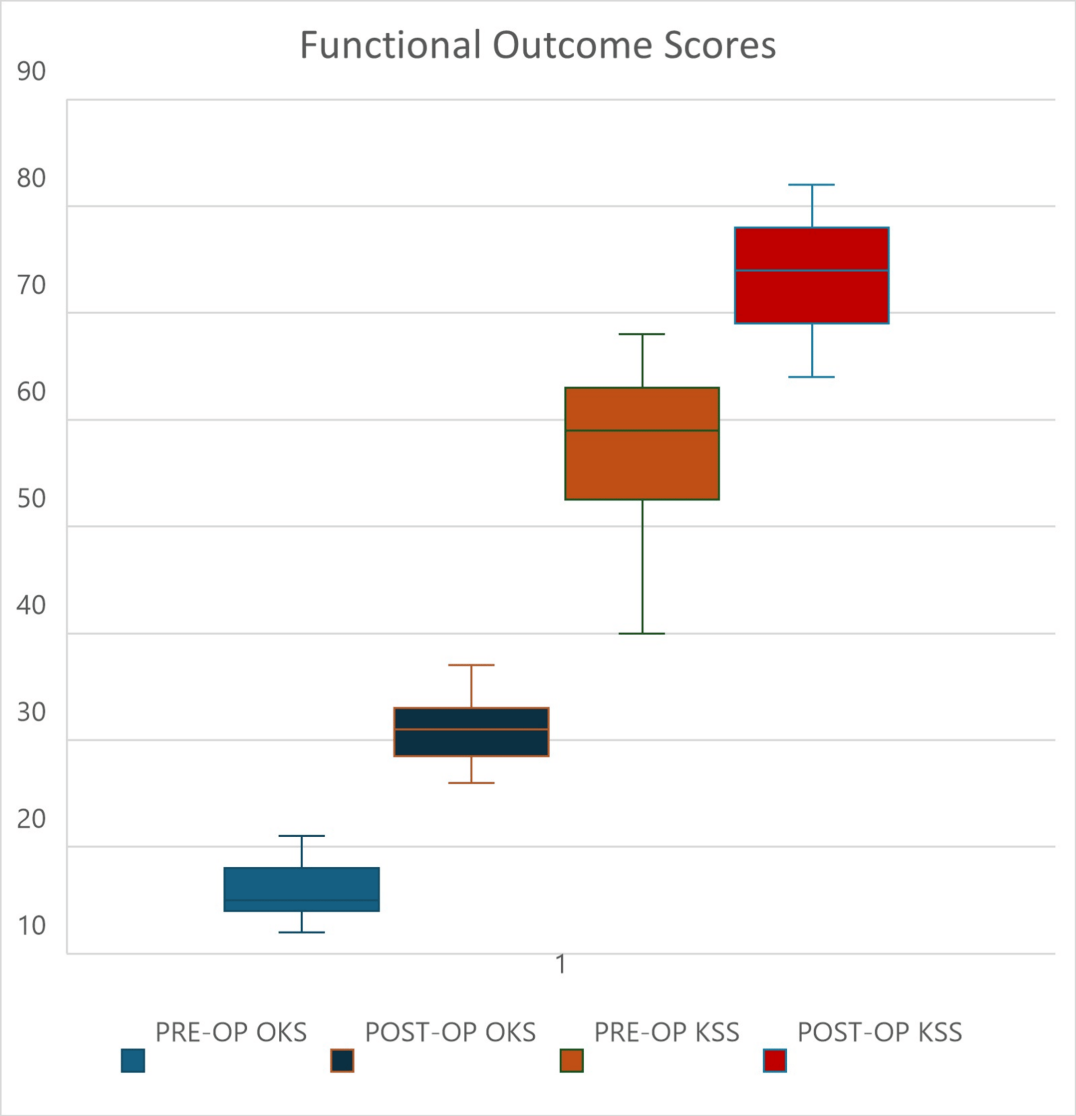
Functional outcome using OKS and KSS. OKS: Oxford Knee Score; KSS: Knee Society Score

Patients demonstrated marked improvements in both pre-operative and post-operative Knee Society Scores (KSS) and Oxford Knee Scores (OKS). The OKS improved significantly from an mean of 15.86 (SD=2.8) to 30.66 (SD=2.8), and the KSS increased from 57.97 (SD=6.9) to 73.59 (SD=4.8), both of which were found to be statistically significant (Figure 3, Table 4). Additionally, the range of motion experienced improvement, with the mean pre-operative flexion increasing from 55.4 degrees to 102.2 degrees by the final follow-up.

**Table 4:** Functional outcome measurement - Oxford Knee Score (OKS) and Knee Society Score (KSS) *Paired student t-test

	Mean	SD	P-Value
Pre-op OKS	15.86	2.84	<0.0001*
Post-op OKS at final follow-up	30.66	2.79	
Pre-op KSS	57.97	6.9	<0.0001*
Post-op KSS at final follow-up	73.59	4.8	

Radiological evaluations conducted during the last follow-up revealed stable positioning and alignment of the components. Additionally, radiographic evidence of osseous integration was observed in all implants, with no signs of component loosening detected.

Complications

None of the patients had any sleeve-related complication. However, tibial stem tip pain was reported in two patients (6.9%), specifically those who received a tibial stem of 115 mm in length. This condition was resolved by the final follow-up, having been managed conservatively using NSAIDs administered as needed for pain alleviation.

Furthermore, four patients (13.8%) experienced post-operative recurrent periprosthetic joint infection (PJI), necessitating debridement and polyethylene exchange (DAIR) along with a 6-week regimen of antibiotics, tailored to the culture and sensitivity results. Subsequently, these patients recovered completely, with no evidence of residual infection.

**Figure 4:**
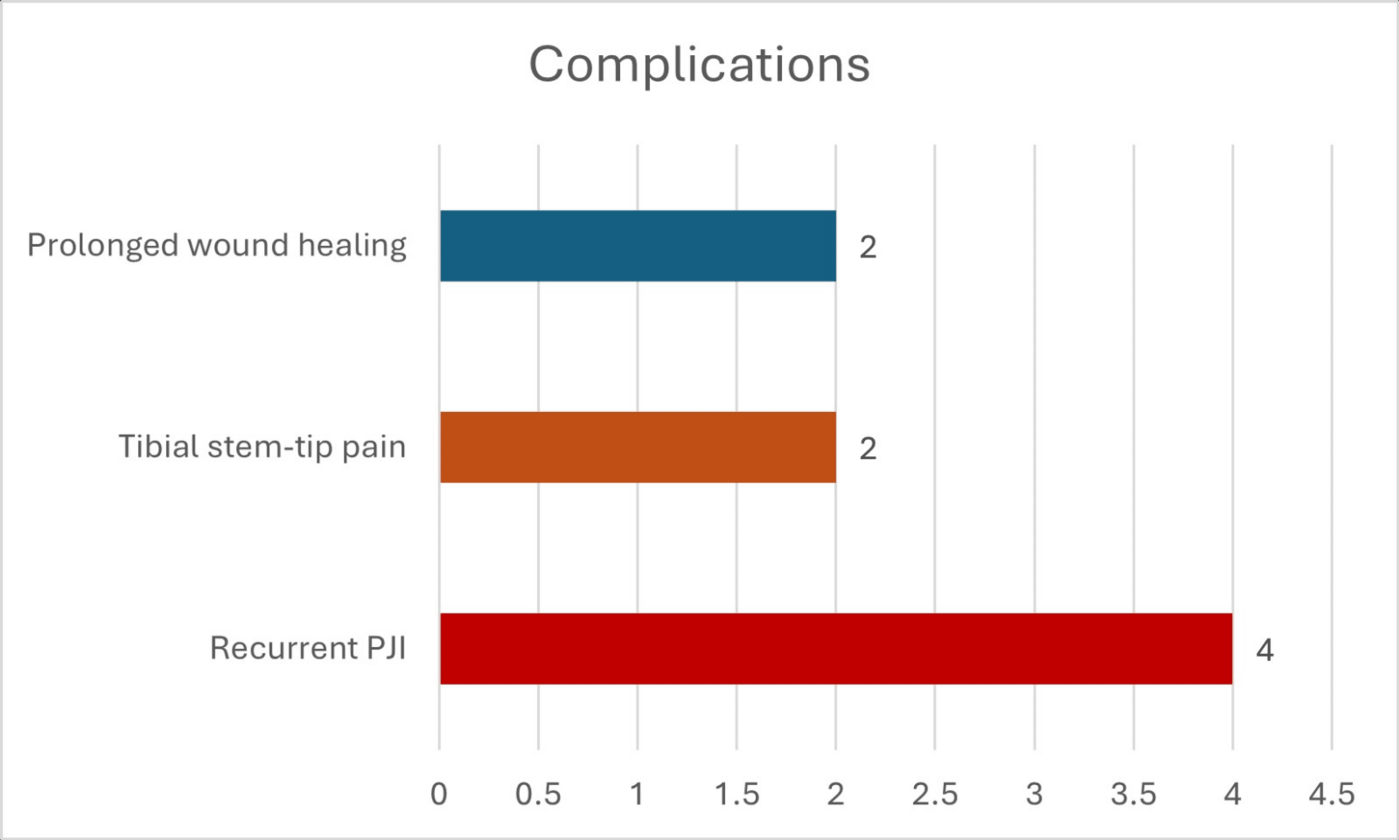
Complications PJI: periprosthetic joint infection

Additionally, two patients (6.9%) had prolonged wound healing and staple removal. These cases were successfully managed through conservative means, specifically with regular dressing changes, and did not require antibiotic therapy (Figure 4).

Survival analysis

**Figure 5:**
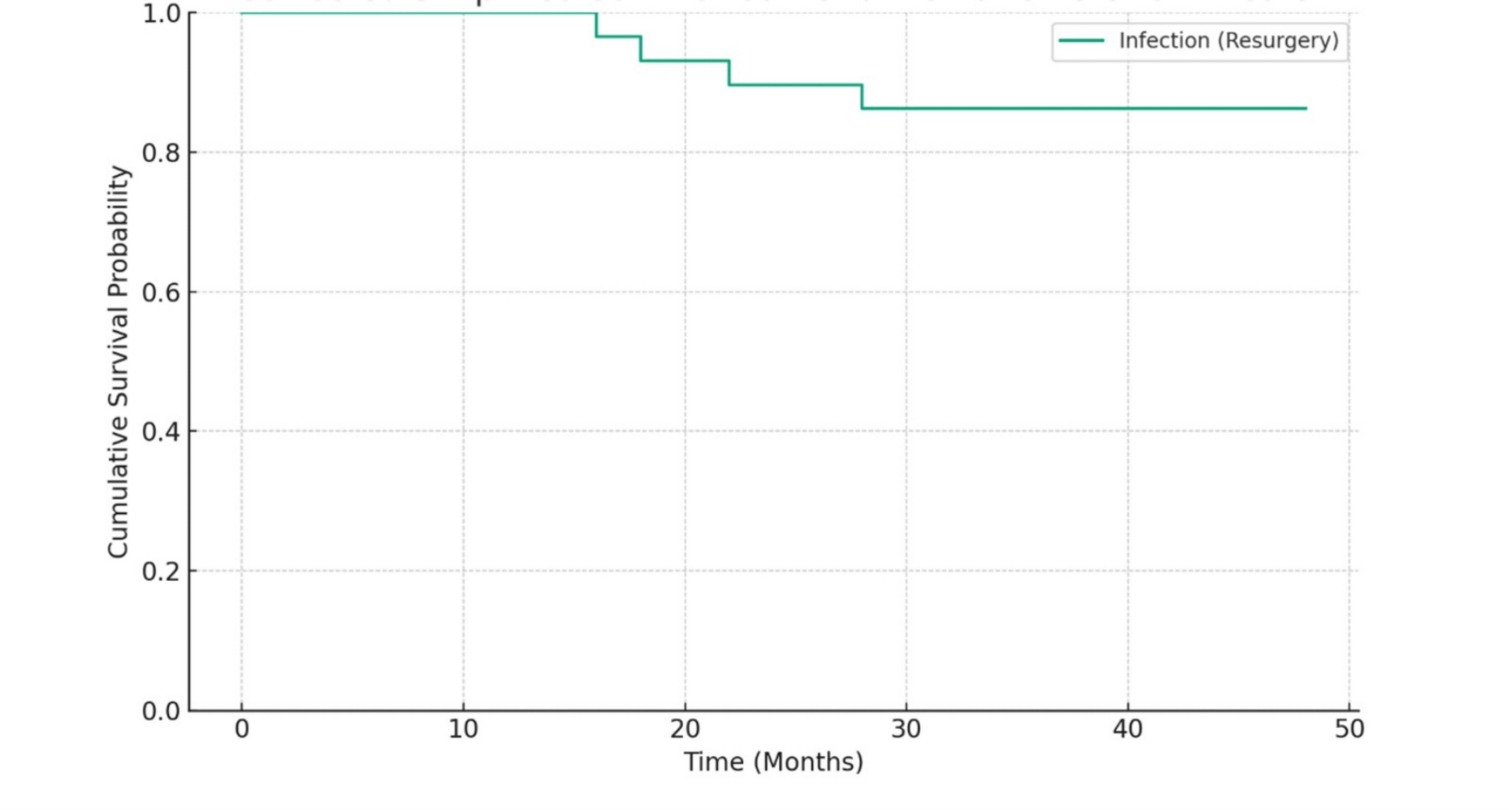
Kaplan-Meier survival analysis with infection (resurgery) as the end-point.

Over the 4-year follow-up period, the Kaplan-Meier survival analysis demonstrates a prolonged period of event-free survival in the cohort of 29 patients with a survival rate of 86.2%. It illustrates a high overall cumulative survival probability, signifying a low incidence rate of the resurgery among the patients throughout the study (Figure 5).

## Discussion

The utilization of metaphyseal sleeves in revision total knee arthroplasty (RTKA) represents a pivotal advancement in the management of complex surgical challenges, including significant bone loss and the need for durable implant fixation. The findings of our investigation underscore the efficacy of metaphyseal sleeves in RTKA, presenting them as a favorable option for achieving optimal surgical outcomes [11-13].

Our research revealed substantial improvements in patient-reported outcomes following the employment of metaphyseal sleeves in RTKA, evidenced by significant increases in the Oxford Knee Score (OKS) and the Knee Society Score (KSS), alongside enhancements in the range of motion. These findings align with the overarching goal of RTKA, which is to alleviate pain, restore knee function, and improve quality of life for patients suffering from the failures of previous knee replacements. The importance of these outcome measures cannot be overstated, as they directly reflect the patient's perception of success following surgery.

The occurrence of recurrent periprosthetic joint infection (PJI) in four patients, who were subsequently successfully treated with debridement, antibiotics, irrigation, and implant retention (DAIR), underscores the persistent challenge of infection management in RTKA. However, the successful resolution of these cases without residual infection highlights the potential of comprehensive treatment strategies in managing such complications.

Our study also noted instances of tibial stem tip pain in two patients, which were managed conservatively. While this complication is not uncommon, its management and the factors contributing to its occurrence warrant further investigation to minimize its impact on patient satisfaction and outcomes.

The literature corroborates the beneficial role of metaphyseal sleeves in addressing the multifaceted issues encountered in RTKA. Mancuso et al. [14] and Chalmers et al. [15] have highlighted the critical function of metaphyseal sleeves in achieving joint line restoration and implant stability, crucial for the long-term success of the revision procedures. The utility of these sleeves in rectifying bone insufficiencies, as emphasized by Dalury and Barrett et al [16]. further supports their adoption in clinical practice.

Additionally, the significance of achieving robust fixation across multiple zones, as discussed by Huff and Sculco, is echoed in our findings TKA [17, 18]. The integration of diaphyseal stems with metaphyseal sleeves to secure fixation in zones 2 and 3 is instrumental in enhancing the overall stability of the implant, a key determinant of the success of revision procedures [19].

The debate between cemented versus uncemented sleeves is a notable aspect of the discourse on metaphyseal sleeves. The findings of Barnett et al. [20] and Graichen et al. [21] advocating for the effectiveness of uncemented sleeves underscore the evolving perspectives on optimal fixation strategies in RTKA. These studies contribute to a nuanced understanding of the advantages and potential limitations of both approaches, informing clinical decision-making.

The exploration of alternative treatments for bone defects, such as autografts and allografts, reveals the complexities of managing AORI types 2A/2B or 3 defects. The discussions by Vasso et al. [6] and Daines and Dennis [22] on the challenges associated with allografts, including availability issues in developing countries and the risks of disease transmission, underscore the need for reliable alternatives. In this context, metaphyseal sleeves emerge as a compelling option, offering dependable fixation without the associated risks of allografts [23].

However, the retrospective nature of our study and the absence of a control group present inherent limitations that may introduce biases into our findings. The potential impact of these limitations on the results warrants careful consideration, underscoring the need for cautious interpretation of the data. Furthermore, the duration of follow-up in our study may not adequately capture the long-term outcomes and survivorship of the metaphyseal sleeves, an aspect that is critical to the comprehensive evaluation of their efficacy and durability.

Future research should aim to address these limitations through prospective studies with larger cohorts and extended follow-up periods. Such studies would provide more robust evidence on the long-term performance of metaphyseal sleeves in RTKA, contributing valuable insights into their role in managing complex revision cases. Additionally, further investigations into the specific factors influencing patient outcomes, such as the management of tibial stem tip pain and the optimization of fixation strategies, would enhance the understanding and application of metaphyseal sleeves in clinical practice.

## Conclusions

Metaphyseal sleeves offer a viable solution for managing severe bone loss in RTKA, providing stable fixation, restoring joint line kinematics, and facilitating stress distribution to the metaphyseal region. This study corroborates the effectiveness of metaphyseal sleeves in challenging revision scenarios, aligning with previous research. Further studies with larger patient cohorts and longer follow-ups are needed to validate these findings.
